# The Service of Meals in Asylums

**Published:** 1892-08-27

**Authors:** 


### ASYLUM ADMINISTRATION.

### THE SERVICE OF MEALS IN ASYLUMS.

(By a Medical Superintendent.)

This is a question which receives too little attention at the hands of Commissioners and superintendents. They have never fully realised how dreary at the best is the monotony of asylum existence, and how eventful the meal hours are in that existence. If one thinks for a moment of the slow and tedious hours of a long voyage, and how the passengers count even the minutes until meal-time, one may realise how much more it is to the inmates of asylums, deprived of their liberty and confined to the dreary routine of months or years. That being so, it is worth while considering what can be done to make this portion of their existence—the time spent at meals—as pleasant and enjoyable as possible. Let us first take the site of the dining-hall in the structure of asylums. Is it placed in an open situation with plenty of light, a good outlook, and cheerful surroundings? Let anyone who knows several asylums think out this question, and his answer must, in many cases, be "No." Many of our dining-halls are placed in a well of the building, flanked by walls on every side, and because through the windows a view of nature is impossible, the miserable substitute of stained glass, skylights, or side windows is introduced instead. This is a fundamental error in the position and construction of dining halls. The softening influence of a free outside atmosphere, abundance of light and ventilation, and a view of nature (not stone and lime) are lost to minds and senses still susceptible to these advantages. Next let us consider the arrangements of the tables in the dining-halls. As a rule, for want of space they are crowded together, and the patients are packed alongside of them with a minimum of elbow room. The tables are too long, and too many patients are seated under the service of each attendant, with a result that you have some of the worst evils of the barrack system, a system by no means calculated to tone down explosive tendencies or to promote the general comfort and well-being of the patients.

Dining in association, which is so highly commended by Commissioners in England and Scotland, and which is only an advance of quite recent years, is very commendable in the the abstract, as compared with dining in the wards, which has many obvious disadvantages. But the aggregation in one immense dining-hall, in full view at the same moment of hundreds of insane people closely packed, feeding untidily, and in many objectionable ways, is a wholesale exhibition of lunacy in a decidedly disagreeable form. We have not yet got the length of breaking up our dining-halls so as not to see the whole scene at once, so as to classify and individualise at the same time, and to break down the barrack system as it at present exists. The table appointments in many asylums are often positively filthy. Tablecloths if changed every morning would still be filthy and objectionable by dinner time. At Larbert Asylum marble tops have been introduced to obviate the use of soiled tablecloths. We do not know how this change will commend itself to patients who have been accustomed all their lives to something warmer and more furnished-looking than a dead cold marble slab, and for the sake of having a cloth some may prefer the dirt after all. At Bothwell Asylum white waxcloth has been used for a number of years with excellent results; but certainly the ideal, if it could be attained, is a snow-white table-cloth. We commend the consideration of this question to superintendents and nurses. Much difference of opinion and practice prevails regarding the utensils placed on asylum dining-tables, and even now we are still bound by cast-iron fetters and the traditional fear of what the insane may unexpectedly do. Glass tumblers are nicer than metal ones; sharp knives are not really more dangerous than blunt ones, and they are less exasperating to irritable patients, whilst no more suggestive of suicide.

To come now to the rules for the serving of meals: these are in many instances conspicuous by their absence, only general principles being laid down. Many attendants and nurses will not take the trouble to work out these principles into specific rules and regulations. The first important point to be aimed at is punctuality and order in reaching the dining-hall; next, perfect stillness and decorum during the saying of grace before meat, and a distinct pause between each course. We have seen in asylums the greatest indecorum, straggling patients and officials coming into the hall while grace was being said, some attendants in so great a hurry to get the meals started that they could not wait for the "Amen," others pushing ahead with the second course before their neighbours were done with the first, and at the same tables some patients feeding hurriedly and ravenously at the second course whilst others were being allowed to neglect or drag slowly along the first. We do not hesitate to say that a wellmanaged asylum, contented and mannerly patients, are as much the results of the discipline and liberality of the dining-hall as of any other part of asylum administration. We are disposed to go further, and to express the conviction that an asylum dietary of most varied character and physiologically nutritive, combined with a cheerful and happy service of meals, is a factor second to none in the general treatment and management of the insane.

One other matter of importance—though almost super-human obstacles block the way in large asylums—is the association of the sexes at the same tables at meals. To be deprived of one's liberty and placed in an asylum means a great deal more than appears at first sight. A certain amount of liberty is imperative. Liberty to do what? A hundred things—the privilege of doing which we do not realize until we are forbidden to do them. Why debar the two sexes so absolutely from association with each other? Why not allow the more rational insane of both sexes to meet daily *en famille* or at *table d'hote?* In two Scotch asylums this has been done, on a small scale at Haddington, on a larger scale at Bothwell, and in both cases with excellent results. It is neither wise nor right to stifle natural and legitimate human cravings. At some date, not too far distant, we may hope that even those whose illness is such as to put in abeyance their reason, will still be recognised as afflicted men and women, and treated accordingly.

